# Focused Assessment with Sonography in Trauma for Assessment of Injury in Military Settings: A Meta-analysis

**DOI:** 10.4274/balkanmedj.galenos.2019.2019.8.79

**Published:** 2019-12-20

**Authors:** Xingshun Qi, Jing Tian, Rui Sun, He Zhang, Jinsong Han, Hai Jin, Hui Lu

**Affiliations:** 1 Military Medical Research Group, General Hospital of Northern Theater Command Shenyang, Liaoning Province, China; 2 Meta-Analysis Interest Group, Department of Gastroenterology, General Hospital of Northern Theater Command, Shenyang, Liaoning Province, China; # Co-first authors.; ★ Co-corresponding authors.

**Keywords:** Focused assessment, injury, military medicine, trauma, ultrasound

## Abstract

**Background:**

Non-invasive, rapid, and precise assessment of injury in the military settings is extremely important, yet difficult. Focused assessment with sonography in trauma (FAST) is being increasingly employed for assessing the location and severity of injury and guiding further treatment strategy. However, the evidence regarding the utility of FAST in the military settings is scattered.

**Aims:**

To evaluate the diagnostic performance of FAST in the assessment of injury in the military settings.

**Study Design:**

Meta-analysis.

**Methods:**

We identified all relevant papers via the PubMed, EMBASE, and Cochrane Library databases. We evaluated the quality of included studies by the Quality Assessment of Diagnostic Accuracy Studies-2 tool. We pooled the area under the curve (AUC), sensitivity, specificity, positive and negative likelihood ratios, and diagnostic odds ratio as the effect sizes, followed by evaluating the heterogeneity among the studies by p value and I^2^.

**Results:**

Among the 39 papers, a total of six papers were included. The sample size ranged from 15 to 396. The AUC of FAST for assessing the injury was 0.85. The pooled sensitivity, specificity, positive and negative likelihood ratios, and diagnostic odds ratio were 0.66, 0.98, 33.1, 0.34, and 97, respectively. The heterogeneity among the studies was statistically significant (p=0.006, I^2^=78%).

**Conclusion:**

FAST is potentially valuable for assessing injury in the military settings. Due to its high specificity, FAST may be appropriate to rule in significant injury. However, because of its poor sensitivity, the ability of FAST to rule out injury cannot be relied upon.

In the military settings, rapid and precise assessment and management of injury need to be warranted to avoid the progression of injury and death; however, it is very difficult because the injury is usually complicated and severe, with the situation being worsened because of limited source of diagnostic instruments. It has been reported that patients with abdominal penetrating wounds should have an exploratory laparotomy as soon as possible (1). During the World Wars I and II, all soldiers with abdominal gunshot wounds underwent a routine laparotomy (2). Since Shaftan questioned its nature of over-treatment for the first time (3), several studies (4,5,6,7,8,9), including the Vietnam wound analysis (10), have supported the possibility of “negative laparotomy,” “unnecessary laparotomy,” and/or “non-therapeutic laparotomy.” However, a non-invasive identification of a “negative or unnecessary laparotomy” is not easy. Nowadays, military radiological techniques are being increasingly employed to assess the location and severity of injury (11). Focused assessment with sonography in trauma (FAST), a hand-held point-of-care ultrasound, seems to be the most frequently used front-line imaging modality (12). Additionally, the implementation of FAST does not need specialized radiologists who are often lacking in role 1 and 2 military facilities (13). More importantly, FAST can effectively classify the causalities into three major types that are useful for guiding further treatment strategy. These types are: (1) negative injury in those needing further clinical observations; (2) suspected injury in those needing further imaging observations; and (3) positive injury in those needing immediate surgery. However, the evidence regarding utility of FAST in the military settings is scattered and inconclusive. Herein, we collected all available evidence and combined the relevant data to explore the diagnostic performance of FAST at assessing injury in the military settings.

## MATERIALS AND METHODS

This work was performed according to the Preferred Reporting Items for Systematic reviews and Meta-Analyses statement (14) and registered in the PROSPERO, with registration number CRD42019134305.

### Search strategy

PubMed, EMBASE, and Cochrane Library databases were searched for relevant papers since the inception of these databases. The last search date was June 2, 2019. The search items were as follows: (FAST) AND [(Combat) OR (War) OR (Military)] AND [(Injury) OR (Trauma)]. Publication language and date were not restricted. All papers regarding the diagnostic performance of FAST at assessing injury in military settings were potentially eligible. Papers were excluded if (1) they were duplicates, case reports, comments, editorials, reviews, or conference meeting reports, (2) they did not employ FAST, or (3) they neither evaluated the diagnostic accuracy of FAST nor extracted the sensitivity or specificity data.

### Data extraction

The data were extracted by the first author as follows: first author, journal, publication year, sources of patients/causalities, period of enrollment, FAST machine and view areas, reference standards for assessment of injury, characteristics of trauma, number of patients/causalities undergoing FAST and those with positive and negative injury via FAST, and reference standards. If there was any uncertainty, he would discuss with others and reach a consensus.

### Study quality assessment

The Quality Assessment of Diagnostic Accuracy Studies-2 (QUADAS-2) tool was employed to assess the methodological quality of included studies (15). This tool assesses the risk of bias by answering the signaling questions in four domains (patient selection, index test, reference standard, and flow and timing) and the applicability concerns by answering questions in the first three of the four domains. Studies with more “low risk of bias” and “high applicability concern” would be of higher quality. Review Manager version 5.3 (Copenhagen: The Nordic Cochrane Centre, The Cochrane Collaboration, 2014) was employed to draw the schematic diagram. The study quality was evaluated by the first author, and in case of difficulty, a consensus was reached through a discussion with other authors.

### Statistical analysis

True positive, false positive, true negative, and false negative values were extracted from the original papers into a table. The “Midas” module in the Stata/SE 14.0 for Windows (SataCorp LP, TX USA) was employed to perform all meta-analyses. Area under the curve (AUC), sensitivity, specificity, positive and negative likelihood ratios, diagnostic odds ratio, and post-test probability were calculated. Their 95% confidence intervals (95% CIs) were calculated, if any. Heterogeneity was evaluated by chi-square and inconsistency tests. P value and I^2^ were calculated. If p value was <0.1 and/or I^2^ was >50%, the heterogeneity would be statistically significant. Heterogeneity was also visually evaluated by the Galbraith plot. Neither meta-regression nor publication bias analysis was performed due to a relatively small number of included studies.

## RESULTS

### Study Selection

**Figure 1:**
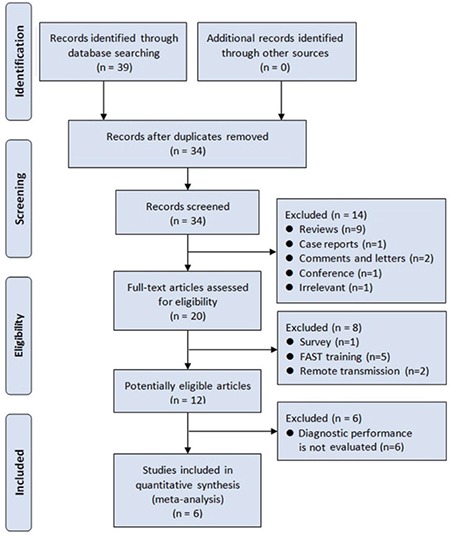
Flowchart of study inclusion.

Among the 39 papers retrieved, a total of 6 papers were finally included (Figure 1) (16,17,18,19,20,21). The study characteristics are shown in Table 1. They were published between 2005 and 2019. The data were reported from Iraq, Afghanistan, South Africa, and Saudi Arabia. The FAST machine was different among the studies. Four regions including pericardial, perihepatic, perisplenic, and pelvic were detected via the FAST. Reference standards for assessing the injury included computed tomography (CT), follow-up observation, and/or surgery. The sample size ranged from 15 to 396.

### Study quality

**Figure 2:**
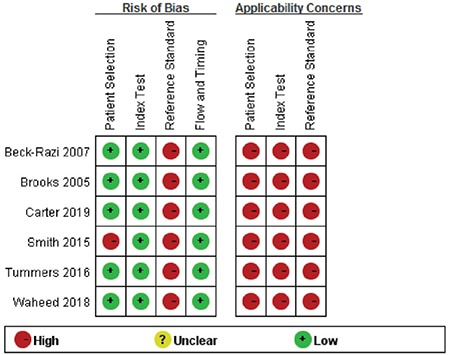
Diagram of study quality assessment.

The study quality is shown in Figure 2. For the signaling question “Was a consecutive or random sample of patients enrolled?” in the “patient selection” domain, one study had an answer of “no,” because it mentioned “During the initial assessment of casualties in this study, FAST was performed on 398 of 468 (85.0%) casualties” and “A total of 403 of 468 (86.1%) casualties in the study group had abdominal/pelvic CT.” For the signaling question “Did the study avoid inappropriate exclusions?” in the “patient selection” domain, one study had an answer of “no,” because it mentioned “Reports were unavailable in 2 cases.” For the signaling question “Were the reference standard results interpreted without knowledge of the results of the index tests?” in the “reference standard” domain, all included studies had an answer of “no,” because the reference standard tests were performed after FAST in all the studies.

### Meta-analyses

**Figure 3:**
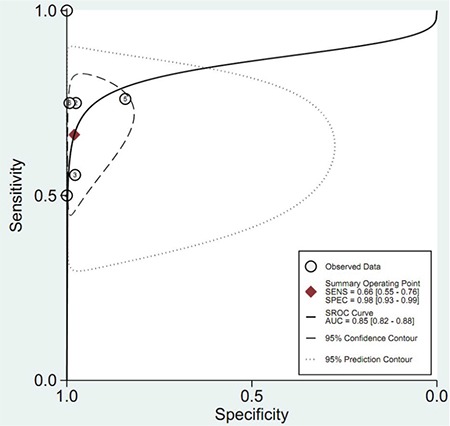
Summary of receiver operating characteristic plot of focused assessment with sonography in trauma for assessing injury.

The AUC of FAST for assessing injury was 0.85 (95% CI=0.82-0.88) (Figure 3).

**Figure 4:**
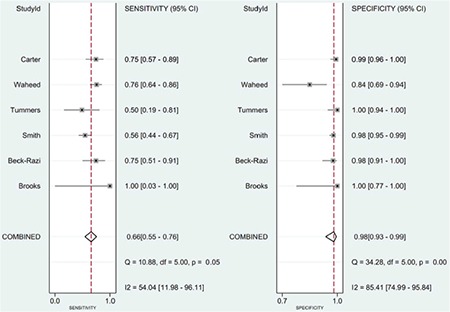
Summary of sensitivity and specificity of focused assessment with sonography in trauma for assessing injury.

The pooled sensitivity was 0.66 (95% CI=0.55-0.76) and specificity was 0.98 (95% CI=0.93-0.99) (Figure 4).

**Figure 5:**
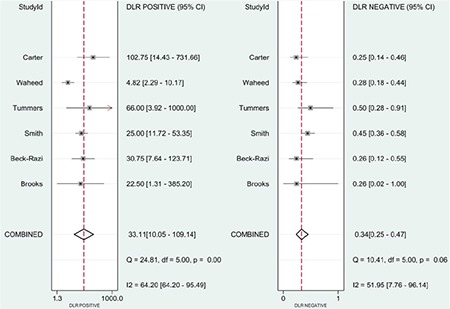
Summary of positive likelihood ratio and negative likelihood ratio of focused assessment with sonography in trauma for assessing injury period.

The positive likelihood ratio was 33.1 (95% CI=10.0-109.1) and the negative likelihood ratio was 0.34 (95% CI=0.25-0.47) (Figure 5).

**Figure 6:**
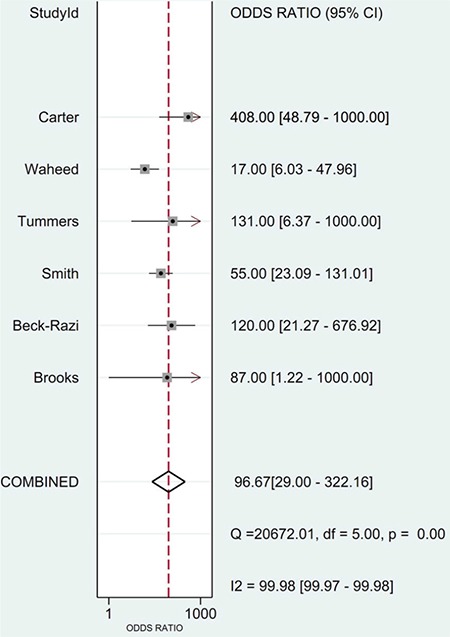
Summary of diagnostic odds ratio of focused assessment with sonography in trauma for assessing injury.

The diagnostic odds ratio was 97 (95% CI=29-322) (Figure 6).

**Figure 7:**
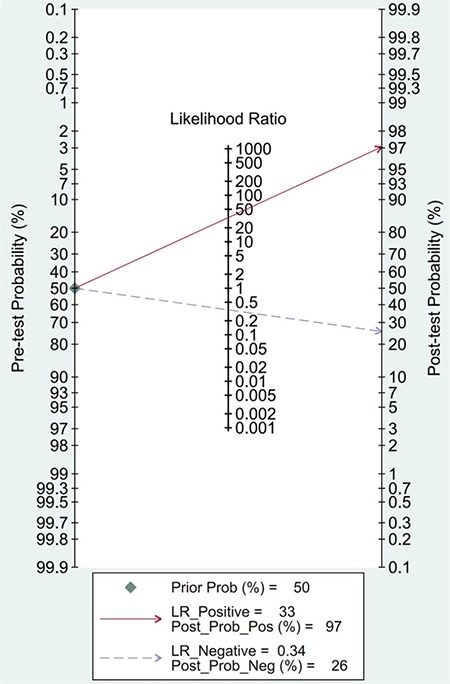
Post-test probability of focused assessment with sonography in trauma for assessing injury.

If FAST was positive, the post-test probability could be estimated as 97%, and if negative, the post-test probability could be estimated as 26% (Figure 7).

### Heterogeneity

The heterogeneity among the studies was statistically significant [p=0.006, I^2^=78% (95% CI=51%-100%)]. Galbraith plot with true positive rate as an effect indicator suggested all studies to be within the 95% CI. Galbraith plot with true negative rate as an effect indicator suggested that the study by Waheed et al. (20) was not within the 95% CI.

## DISCUSSION

To the best of our knowledge, our study is the first meta-analysis to explore the role of FAST in assessing injury in the military settings. Several previous systematic reviews and meta-analyses regarding application of FAST have been fully acknowledged (22,23,24), and their features have been compared with our study. First, our study, rather than previous work, focused on the military settings. It should be noted that the severity and complexity of injury and accessibility of diagnostic equipment and therapeutic modalities are greatly different in civilian and military settings. Second, Quinn’s study performed a systematic literature review (22), but did not combine the data by means of a meta-analysis. Third, among Stengel’s papers, one focused on the patients with blunt abdominal trauma (23) and another on the patients with blunt thoracoabdominal trauma (24). Quinn’s paper focused on the patients with penetrating torso trauma (22). By comparison, our present study did not limit the type of injury. Fourth, Stengel’s study regarding blunt abdominal trauma identified 4 papers (23) and that regarding blunt thoracoabdominal trauma identified 34 papers (24), and Quinn’s study regarding penetrating torso trauma identified 8 papers (22). By comparison, our present study identified 6 papers.

Our study found that FAST had a moderate diagnostic accuracy with an AUC of 0.85 and a very high specificity (i.e., true negative rate) of 0.98, but a relatively low sensitivity (i.e., true positive rate) of 0.66. These findings suggested that the performance of FAST at identifying patients with severe injuries may be moderate based on an undesired sensitivity value, and some patients with truly severe injuries may be missed by FAST. But its performance at identifying patients who did not have severe injury was very high, and only few patients were misdiagnosed with severe injury. This may be translated into our clinical practice. Thus, if FAST indicates a positive finding, we can be confident that it is positive. In other words, we can rule in the injuries that we are looking for, if FAST shows a positive finding. Certainly, the heterogeneity of the available studies should not be neglected to precisely recognize the potential limitation of the results.

According to the QUADAS-2 tool for assessing the risk of bias (15), the reference standard domain was at a high risk of bias for all the included studies. However, considering the order of tests, invasiveness of reference standard (i.e., surgery), and nature of study population in real-world practice (i.e., injuries needing immediate management in the military settings), it was impossible to interpret the reference standard results without the knowledge of the results of the index tests. FAST is an easy-to-access and non-invasive index test and firstly performed for the causalities. Then, CT, follow-up observation, and/or surgery, which are considered as the reference standards, are performed to further identify the injury severity.

There was a statistically significant heterogeneity among the studies. This can be explained by the difference in the characteristics of study population. In Tummers’s study, only young children aged <18 years old were selected (19). By contrast, Waheed’s study included only adults aged >14 years (20). In other studies, age was not a limiting factor. Furthermore, the mechanisms of injury may be another source of heterogeneity. In Tummers’s and Waheed’s studies, only blunt abdominal injury was selected (19,20). By comparison, in the other studies, the mechanisms of injury were not clearly restricted. For examples, in Smith’s study, more injured patients had explosive injury and gunshot wounds (18); in Beck-Razi’s and Brook’s studies, patients had blunt and penetrating injury (16,17).

Our study had several limitations. First, the number of included studies was relatively small. Thus, the meta-regression, publication bias, and subgroup analyses could hardly be performed. Additionally, the present results need to be confirmed with large-scale studies. Second, there was a difference in the age and source of target population, mechanisms of injury, and reference standards among the studies. Therefore, the conclusions need to be validated in different settings. Third, we evaluated all injuries in the military settings, rather than focusing on the combat-related injured soldiers alone. There were three studies conducted in hospitals where a majority of injured civilians were admitted. One was from a Level 1 hospital in Lebanon, one from the Red Cross War Memorial Hospital in Cape Town, and another from a Level 1 hospital in Saudi Arabia. Fourth, the type of trauma was not limited. Thus, the results cannot be used for any specific injury. Fifth, only one primary author was initially involved in literature search and data extraction. This behavior was potentially inadequate.

Based on this meta-analysis, FAST has a moderate diagnostic performance at assessing the severity of injury in the military settings. Despite the fact that FAST can rarely misdiagnose a truly positive injury and accurately cover the cases with truly negative injury, it can miss some cases with truly positive injury. The strategies to improve the utility of FAST for identifying more cases with truly severe injury should be explored in future.

## Figures and Tables

**Table 1:**
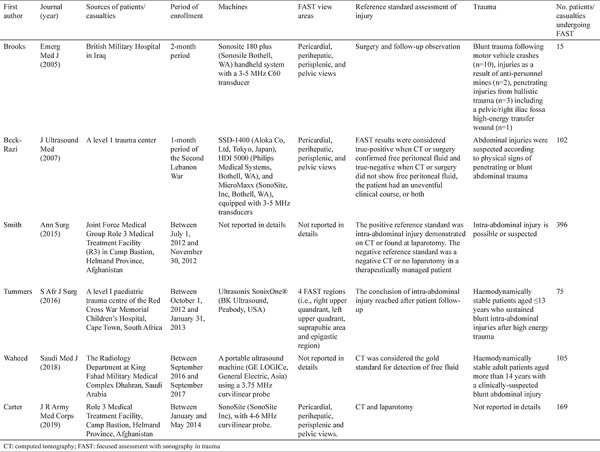
Characteristics of included studies
